# High-frequency ultrasound modulates the interfacial Ni(OH)_2_/NiOOH redox transition to enhance glucose electrooxidation and hydrogen production^☆^

**DOI:** 10.1016/j.ultsonch.2026.108085

**Published:** 2026-09-28

**Authors:** Youness Boukarkour, Christophe Coutanceau, Karine.De Oliveira Vigier, Teko Wilhelmin Napporn, Prince Nana Amaniampong

**Affiliations:** a Institut de Chimie des Milieux et Matériaux de Poitiers, University of Poitiers, CNRS, 1 Rue Marcel Doré, 86073 Poitiers, France; b French Research Network on Hydrogen (FRH2) of CNRS, Nantes, France

**Keywords:** Sonoelectrochemistry, Hydrogen production, Glucose electrooxidation, Biomass valorization, Nickel foam, Alkaline electrolysis

## Abstract

Replacing the kinetically limited oxygen evolution reaction with biomass electrooxidation offers a promising route toward energy-efficient hydrogen production, yet the electrooxidation reaction’s performance remains hindered by interfacial kinetics and catalyst accessibility. Here, we demonstrate that high-frequency ultrasound acts as a dynamic physical field that drives interfacial chemical activation, enabling enhanced glucose electrooxidation on nickel foam under mild conditions. Under acoustic irradiation, the Ni(OH)_2_/NiOOH redox transition undergoes a substantial cathodic shift of approximately 450 mV, leading to an earlier onset of glucose electrooxidation and a significant increase in anodic current density. A qualitative comparison with low-frequency ultrasound (20 kHz), performed as a hydrodynamic benchmark, showed no corresponding shift in onset potential, highlighting an interfacial activation mechanism specific to HFUS rather than a general hydrodynamic effect. As a result, glucose electrooxidation currents increase by up to fourfold at steady state, accompanied by a twofold sustained enhancement in cathodic hydrogen production. We propose that acoustic cavitation dynamically perturbs the electrode–electrolyte interface, increasing electrochemical accessibility and facilitating OH^–^ adsorption. This accelerates the formation and turnover of NiOOH active sites while mitigating surface deactivation. These findings establish acoustic cavitation as a versatile physical lever to enhance interfacial charge-transfer kinetics in transition-metal-mediated biomass electroreforming, offering a strategy complementary to traditional catalyst design and opening new directions for biomass electrooxidation and hydrogen production.

## Graphical abstract

**Figure:**
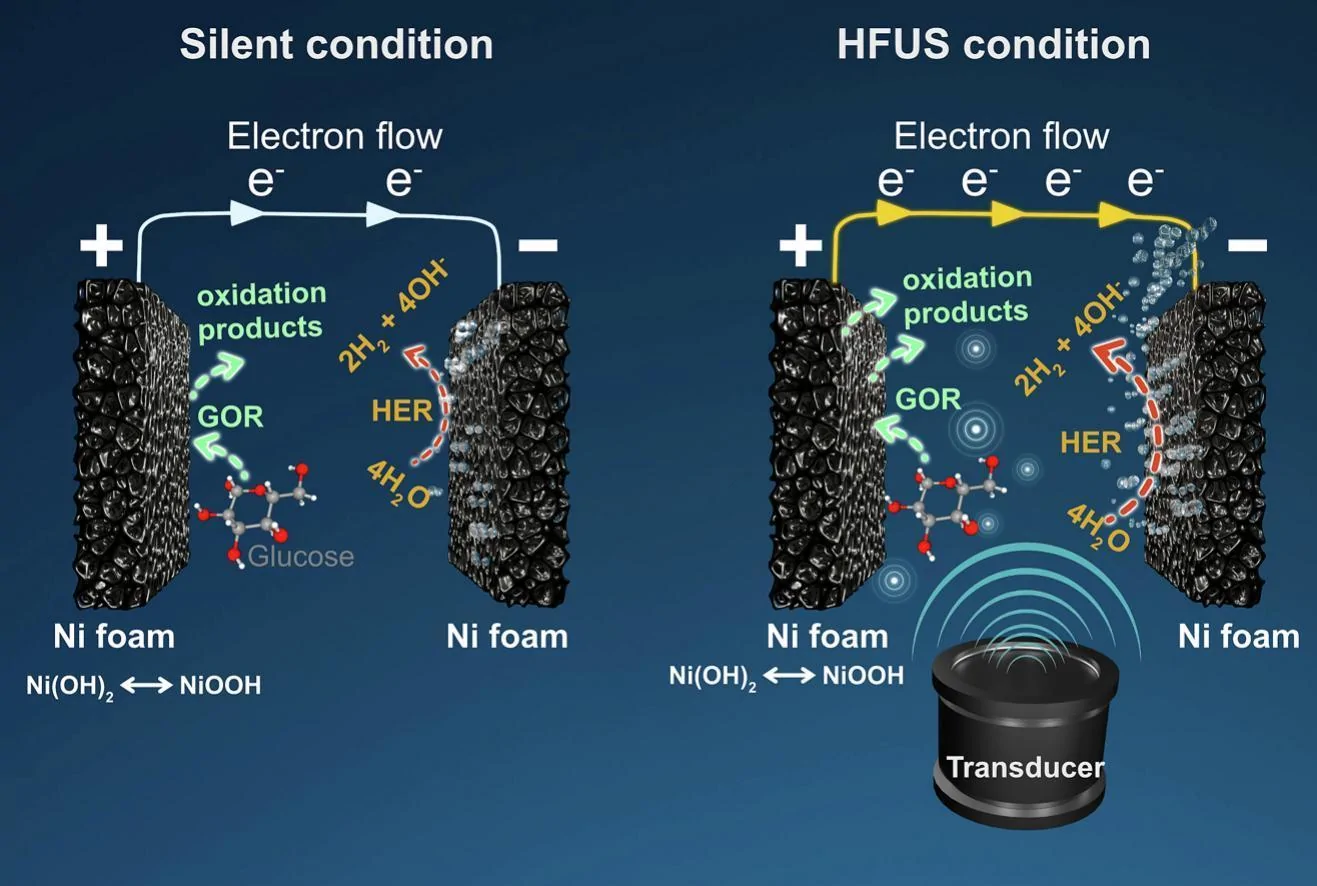

## Nomenclature

HFUS: High-frequency ultrasound (584 kHz in this study)
LFUS: Low-frequency ultrasound (20 kHz in this study)
OER/HER: Oxygen evolution reaction/Hydrogen evolution reaction
CV/CA: Cyclic voltammetry/Chronoamperometry
RHE: Reversible hydrogen electrode
j: Geometric current density (mA cm^−2^)
Q: Faradaic charge (C or mC)
FE(H_2_): Faradaic efficiency toward hydrogen (%)
SEM/EDS(EDX): Scanning electron microscopy/Energy-dispersive X-ray spectroscopy
micro-GC: Micro gas chromatograph
ΔE_p,c_: Cathodic peak potential shift (mV)

## 1 Introduction

Hydrogen production via electrochemical water splitting is essential for the transition toward a sustainable economy. However, its industrial scalability is fundamentally constrained by the sluggish kinetics and high overpotential of the anodic oxygen evolution reaction (OER) [1], [2]. Replacing the OER with the electroreforming of biomass-derived molecules offers a highly attractive alternative. This approach lowers the thermodynamic cell voltage, simultaneously coupling hydrogen generation with possibly the production of high-value organic chemicals at the anode [3], [4], [5].

Glucose is a promising biomass-derived substrate for electroreforming. It is naturally abundant, inexpensive, and chemically versatile through a complex oxidation pathway [6], [7]. In alkaline media, glucose electrooxidation is conventionally mediated by non-noble transition-metal catalysts. In particular, nickel-based anodes drive this process through the reversible Ni(OH)_2_/NiOOH redox couple; here, NiOOH acts as the active chemical oxidant while Ni(OH)_2_ remains in the resting state [8], [9], [10]. Traditional catalyst engineering has yielded highly active materials through nanostructuring and composition tuning [11]. However, this approach cannot resolve three intrinsic interfacial bottlenecks: diffusion-limited solid-state Ni(II)/Ni(III) redox turnover [12], restricted electrolyte accessibility within porous electrodes, and rapid surface deactivation caused by strongly adsorbed reaction intermediates [13], [14], These limitations are fundamentally structural and dynamic; because they arise from the non-equilibrium state of the electrode–electrolyte interface under operating conditions, they cannot be fully resolved by catalyst design alone.

External physical fields offer an alternative route to controlling these interfacial dynamics without modifying the catalyst itself. Prominent examples include the use of magnetic fields to induce magnetohydrodynamic flows [15], and localized inductive heating [16]. Among these, acoustic fields (i.e., ultrasound) are uniquely suited for electrochemical systems: the propagation of sound waves triggers acoustic cavitation, generating microbubbles whose collapse creates localized microstreaming, shock waves, and high-shear liquid microjets. These physical phenomena are well established to enhance bulk mass transport [17], [18], accelerate gas-bubble detachment [19] and continuously mitigate surface fouling [20]. In alkaline media, prior sonoelectrochemical studies on nickel-based materials (e.g., Foroughi et al.) have predominantly used ultrasound as an *ex situ* mechanical pretreatment to physically restructure, erode, or reactivate the electrode surface for the OER and the hydrogen evolution reaction (HER) applications [21], [22]. The combination of electrochemical and ultrasonic activation has also proven beneficial for the valorization of biomass-derived compounds on platinum electrodes [23]. Separately, high-frequency acoustic fields have been shown to drive homogeneous, catalyst-free glucose oxidation in bulk water, relying purely on bulk-phase radical sonolysis [24]. Consequently, to the best of our knowledge, it remains unknown whether *in situ* ultrasound can act beyond a simple mass-transport enhancer for electrochemical reactions on Ni electrodes. Specifically, whether acoustic cavitation can directly modulate the electronic and coordination states of the heterogeneous Ni(OH)_2_/NiOOH redox transition to dynamically increase the accessibility of active sites has not yet been systematically investigated.

Here, we address this gap. Using a custom-built, thermally decoupled sonoelectrochemical reactor to isolate acoustic effects from thermal artifacts, we investigated high-frequency ultrasound (HFUS, 584 kHz) and contrasted its electrochemical behavior with conventional low-frequency ultrasound (LFUS, 20 kHz) operating as a hydrodynamic benchmark. These two frequencies generate fundamentally different cavitation regimes: at 20 kHz, transient cavitation dominates, with large bubbles collapsing violently and causing mechanical shockwaves and surface erosion; at 584 kHz, short acoustic cycles favor a stable acoustic regime dominated by continuously oscillating microbubbles rather than transient violent collapses [18]. We hypothesize that this distinction in cavitation regime produces mechanistically distinct electrochemical effects: an interfacial, kinetic activation of the Ni(OH)_2_/NiOOH redox couple under HFUS, as opposed to a purely diffusion-limited transport enhancement under LFUS.

The primary objectives of this work are therefore twofold: (i) to determine whether high-frequency ultrasound can directly modulate the Ni(OH)_2_/NiOOH redox transition, rather than merely accelerating mass transport; and (ii) to establish, through a comparison with low-frequency ultrasound under a physically distinct configuration, whether this interfacial activation translates into enhanced glucose electrooxidation and coupled hydrogen production.

## 2 Results and discussion

### 2.1 Establishing a controlled acoustic reaction environment

Before probing how HFUS affects nickel-based electrodes and glucose electrooxidation, a reactor configuration was first established to decouple acoustic effects from thermal artifacts. Two sonoelectrochemical setups were compared (see Section 4.3): a direct configuration, in which the electrolyte is in immediate acoustic contact with the transducer, and an indirect configuration, where a borosilicate glass barrier separates the electrode compartment from the direct acoustic field (**Fig. S1**). Crucially, the electrolyte volume (50 mL), initial temperature (20.70 ± 1.90 °C), and working electrode geometry (0.50 cm^2^) were kept strictly identical across both configurations, enabling rigorous isolation of thermal and cavitation contributions.

Under direct irradiation, the electrolyte temperature rises rapidly at a rate of 1.62 °C min^-1^, reaching approximately 65 °C. Even with the transducer's cooling system, this rapid heat buildup prevents continuous operation due to the transducer's manufacturer-specified 40 °C safety limit. This rapid and uncontrolled thermal buildup limits the reliability and reproducibility of electrochemical measurements under identical baseline temperatures. In contrast, the indirect configuration exhibits a much lower heating rate of 0.50 °C min^-1^ and successfully stabilizes the reaction environment near ambient temperature at 20.70 ± 1.90 °C (Fig. 1**a**). This stabilization confirms that the indirect setup effectively suppresses bulk convective thermal effects during prolonged ultrasonic runs.

**Fig. 1:**
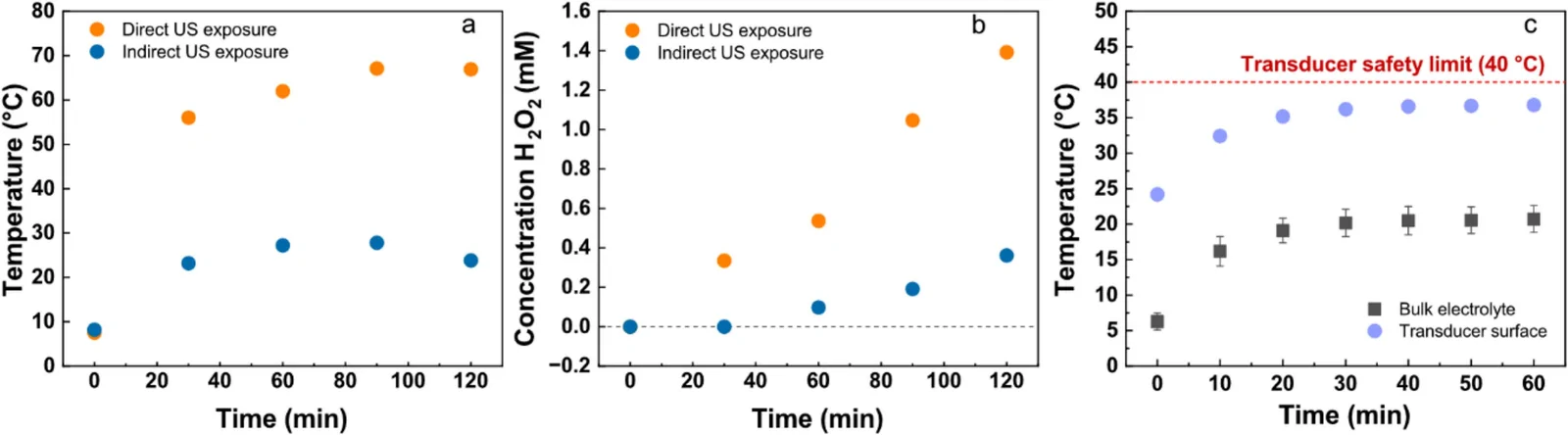
Establishing a controlled acoustic reaction environment. (a) Electrolyte temperature evolution under direct and indirect HFUS configurations (0.10 M NaOH, 584 kHz); (b) H_2_O_2_ accumulation used as a dosimeter for cavitation activity; (c) Bulk electrolyte and transducer surface temperature over 60 min of continuous irradiation at 584 kHz in the indirect configuration, relative to the transducer's 40 °C safety limit (mean ± SD, n = 4 independent measurements on different days; error bars for the transducer surface, SD ≤ 0.7 °C, are smaller than the symbol size).

Achieving this thermal stability comes at the cost of a measurable attenuation of cavitation intensity. We tracked the *in situ* sonochemical formation of hydrogen peroxide (H_2_O_2_) (calculated via Eq. (1)), a widely established probe for monitoring cavitation activity [17], [25], over the first 60 min of irradiation. The generation rate of H_2_O_2_ drops from 8.93 to 1.63 µM min^-1^ when transitioning from the direct to the indirect configuration. After 120 min of continuous sonication, this corresponds to final H_2_O_2_ concentrations of approximately 1.39 mM (direct) and ∼0.36 mM (indirect) (Fig. 1**b**). This attenuation is primarily caused by the partial reflection and absorption of the acoustic wave at the glass-liquid interfaces of the nested cell, which decreases the transmitted acoustic pressure and consequently reduces the total number of cavitation events [18].

While the indirect configuration effectively stabilizes the bulk electrolyte, the transducer itself remains subject to intrinsic thermal buildup during continuous operation, independent of the electrolyte-side thermal decoupling. Over 60 min of continuous irradiation at 584 kHz in the indirect configuration, the bulk electrolyte temperature stabilized at 20.70 ± 1.90 °C, while the transducer surface temperature reached 36.80 ± 0.50 °C, remaining within but close to the manufacturer's 40 °C safety limit (Fig. 1**c**). Even under this thermal margin at the transducer, the bulk electrolyte reaches a stable plateau well within the intended operating window, ensuring that all electrochemical measurements reported in this study were performed under thermally stable and reproducible conditions.

Despite this partial acoustic attenuation, the indirect configuration still generates a measurable and steady accumulation of H_2_O_2_, confirming that the transmitted cavitation activity remains sufficient to drive sonochemical and physical interfacial effects. This combination of a stable bulk temperature and sustained acoustic cavitation makes the indirect configuration well-suited for isolating and probing intrinsic interfacial phenomena. Accordingly, this setup was systematically used for all subsequent electrochemical experiments, unless otherwise stated.

### 2.2 Activating nickel foam as a redox-responsive electrode

Following the selection of the indirect configuration for its thermal stability, the nickel foam electrode was electrochemically activated until stable cyclic voltammograms (CVs) were obtained (**Fig. S2**). We probed the structural and compositional changes induced by this electrochemical conditioning using scanning electron microscopy (SEM) coupled with energy-dispersive X-ray spectroscopy (EDS) (Figs. 2 **and S3**). The as-received foam exhibits partial surface contamination, visible as localized dark spots in the SEM image (Fig. 2**a**), whereas the activated electrode displays a uniformly clean metallic surface (Fig. 2**b**). EDS mapping further reveals a net increase in surface oxygen content of approximately 2.2 at% (**Figs. S4 and S5**), which is highly consistent with surface oxidation and the successful formation of a thin surface hydroxide layer. This electrochemical conditioning triggers the growth of the initial α-Ni(OH)_2_ phase on the metallic nickel ligaments via the interfacial place-exchange mechanism of oxygen and hydroxyl chemisorption at lower polarization potentials (below 0.5 V *vs.* RHE) [26]**.** This α phase subsequently undergoes an irreversible structural transformation into the thermodynamically more stable β-Ni(OH)_2_ phase upon extended cycling to higher anodic potentials [27].

**Fig. 2:**
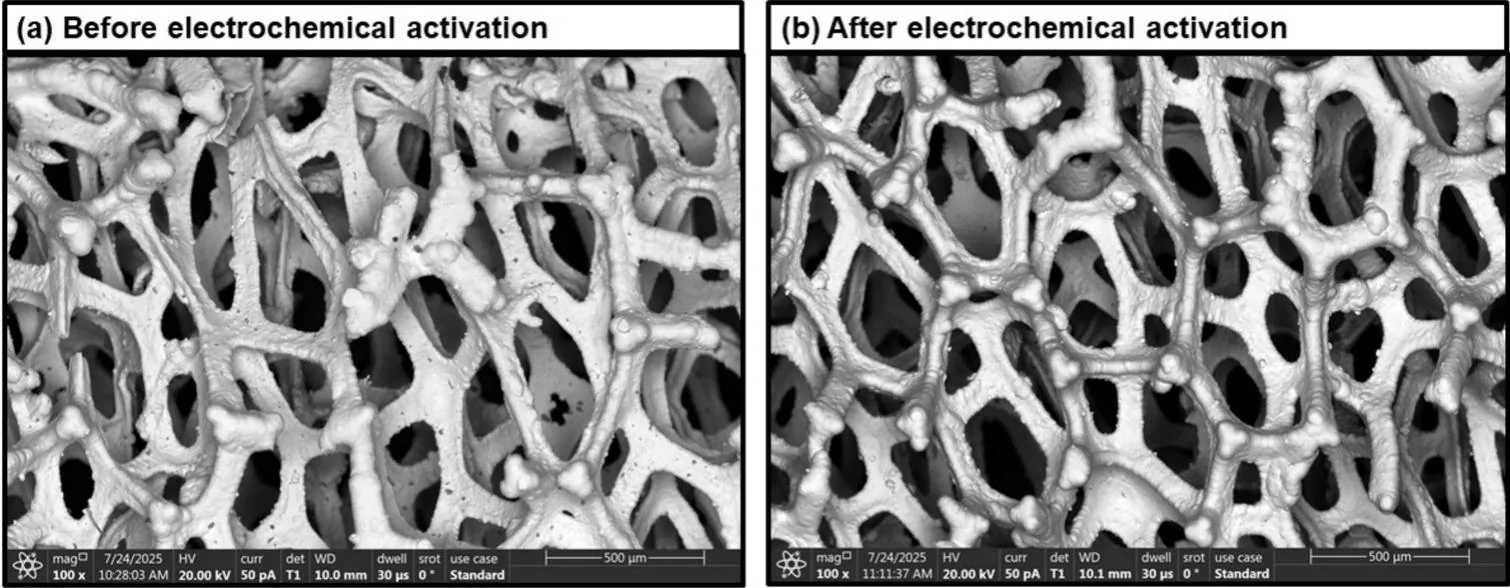
Surface morphology of Ni foam before and after electrochemical activation: (a) SEM image of as-received Ni foam showing smooth ligaments; (b) SEM image of the electrochemically activated Ni foam showing a rougher surface.

The electrochemical impact of this activation was evaluated by cyclic voltammetry (CV) in 0.10 M NaOH under silent conditions (without ultrasonic irradiation). Because electrochemical reactions take place only at the surface of the materials, such an electrochemical method can provide more interesting information than XPS or Raman measurements because only the surface atoms which can be electro-active are probed (the mean escape depth of electrons by XPS is close to 2 nm or more for Ni metal) [28]. Moreover, because the surface electrochemistry of Ni materials is very well known [29], the surface composition can be determined in the environment of its use, which gives access to the real surface structure, whereas *in situ* XPS or Raman measurements give a global multilayer surface composition. In the absence of glucose (Fig. 3**a**), the activated electrode displays a more pronounced Ni(OH)_2_/NiOOH redox response compared to the as-received foam. Specifically, the anodic peak current density reaches 2.46 mA cm^-2^ at 1.48 V *vs.* RHE for the activated electrode, whereas the as-received foam yields only 1.70 mA cm^-2^ at 1.42 V *vs.* RHE. Despite exhibiting nearly identical onset potentials for Ni(OH)_2_ oxidation (∼1.36 V *vs.* RHE), the substantially larger charge capacity associated with the voltammetry peaks of the activated electrode indicates a higher density of electrochemically accessible redox active sites. At more positive potentials (above 1.50 V *vs.* RHE), both electrodes exhibit comparable current densities toward the OER, suggesting that electrochemical conditioning primarily enhances the accessibility of the surface nickel redox couples rather than altering their intrinsic catalytic behavior or Tafel kinetics for water oxidation, in agreement with the dual-path model established by Medrano-Banda et al. [10].

**Fig. 3:**
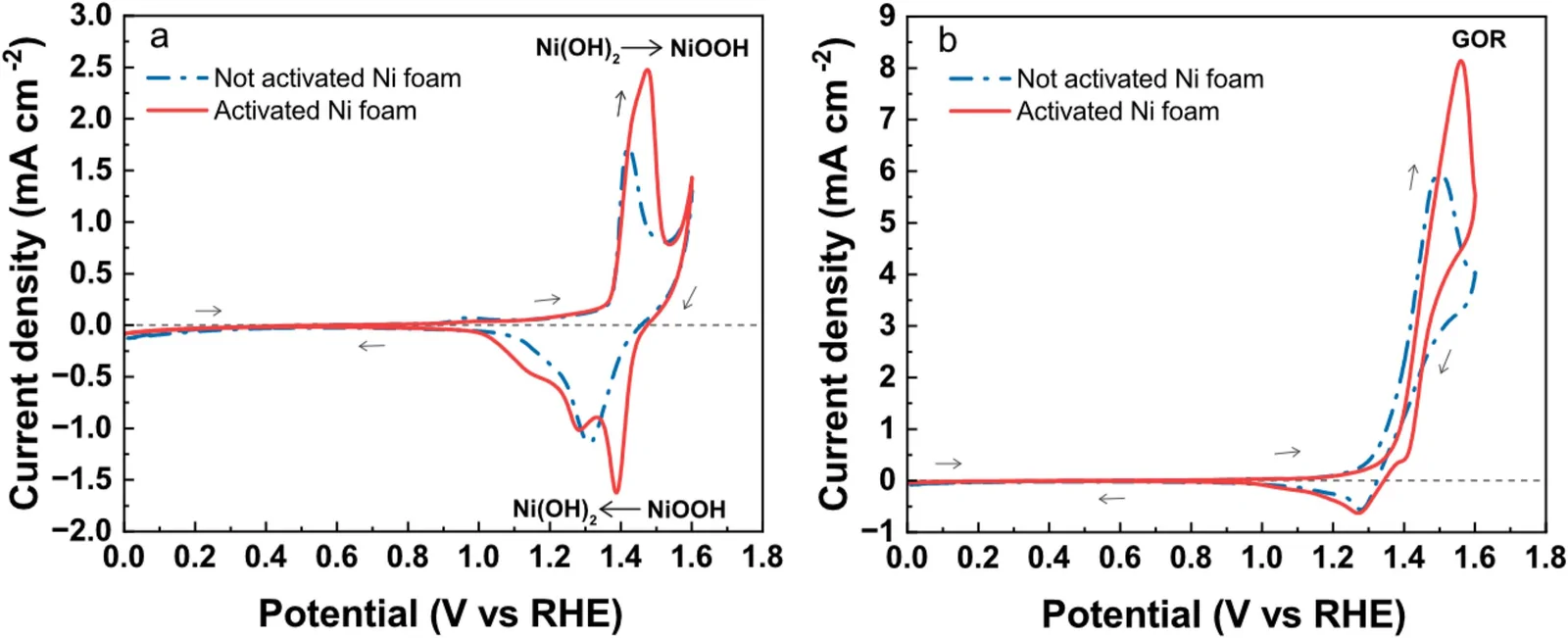
Cyclic voltammograms of as-received and activated Ni foam in 0.10 M NaOH recorded at 10 mV s^−1^ and at room temperature (20.70 ± 1.90 °C): (a) without glucose and (b) with 2.9 mM glucose.

In the presence of 2.9 mM glucose (Fig. 3**b**), the activated electrode achieves substantially higher electrocatalytic current densities than the as-received foam, reaching 8.10 mA cm^-2^ at 1.56 V *vs.* RHE, compared to 5.85 mA cm^-2^ at 1.49 V *vs.* RHE. Both electrodes exhibit a similar catalytic onset potential for glucose electrooxidation at approximately 1.28 V *vs.* RHE. Interestingly, the absence of any further current increase beyond ∼1.50 V *vs.* RHE suggests a kinetic outpacing and masking of the competing OER under these reaction conditions, rather than a permanent suppression of the water oxidation pathways. This behavior arises because the rapid chemical reduction of the electrogenerated trivalent NiOOH phase into the Ni(OH)_2_ phase by glucose continuously regenerates the divalent state, which explains the very low reduction peak intensity at ca. 1.30 V *vs*. RHE in the reverse potential scan, thereby maintaining a very low steady-state surface coverage of the highly oxidized active centers required to drive the sluggish four-electron OER [10]. Taken together, these results demonstrate that electrochemical conditioning enhances the overall glucose electrooxidation activity primarily by increasing the electrochemical accessibility and utilization of the pre-existing NiOOH oxyhydroxide sites within the porous matrix.

### 2.3 Acoustic modulation of Ni(OH)_2_/NiOOH redox chemistry

The influence of HFUS (584 kHz) on the Ni(OH)_2_/NiOOH redox transition was subsequently investigated in the absence of glucose (Fig. 4). Under silent conditions, the CVs display the expected reversible redox behavior, with matched anodic and cathodic charge densities (∼14 mC cm^-2^). Beyond these redox peaks, the onset of the competing OER occurs above 1.50 V *vs.* RHE. Upon acoustic irradiation, the electrochemical response undergoes a dramatic transformation. The anodic current begins to rise sharply at a significantly lower onset potential of approximately 0.90 V *vs.* RHE, while the apparent onset of the OER shifts earlier (∼1.40 V *vs.* RHE). Concurrently, the anodic charge density increases significantly to ∼24 mC cm^-2^, whereas the corresponding cathodic charge density decreases slightly to ∼12 mC cm^-2^. This voltammetric charge asymmetry indicates a substantially enhanced oxidation of Ni(OH)_2_ to the active NiOOH phase under the high-frequency acoustic field, accompanied by a partial loss of electrochemical reversibility.

**Fig. 4:**
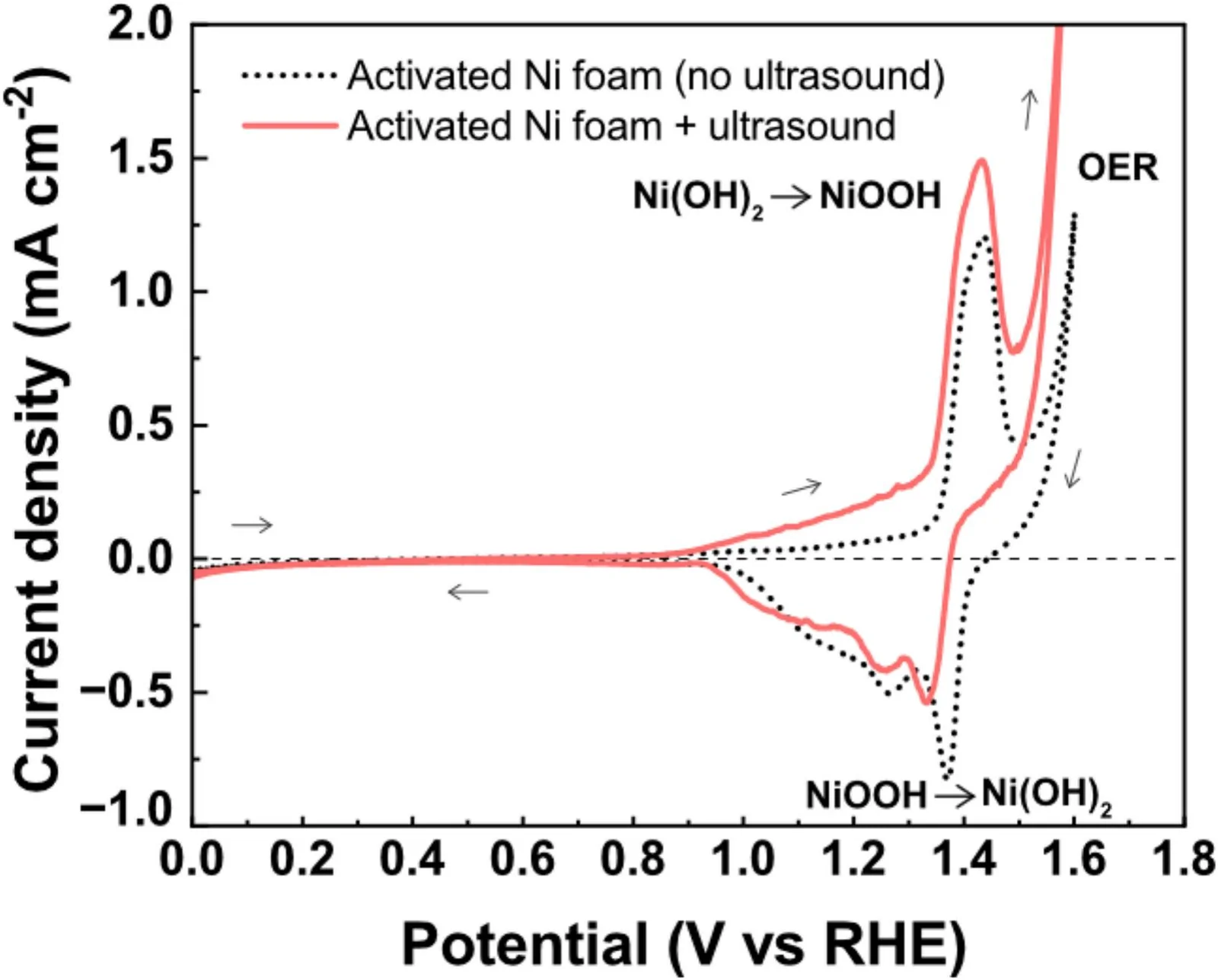
Effect of ultrasound on the Ni(OH)_2_/NiOOH couple in 0.10 M NaOH during the cyclic voltammetry recorded at 10 mV s^−1^ and at 20.70 ± 1.90 °C.

By directly comparing the anodic faradaic charges obtained with and without sonication, we estimate a ∼71% increase in the amount of active NiOOH sites formed under HFUS (Eq. (2), applied to faradaic charge rather than current density). The surface oxidation occurs according to the following equation (Eq. (4), whereas that for the surface reduction according to equation (Eq. (5):

(4) Ni(OH)_2_ + OH^-^ → NiOOH + H_2_O + e^-^

(5) NiOOH + H_2_O + e^-^ → Ni(OH)_2_ + OH

The larger anodic charge relative to the cathodic charge is consistent with enhanced OH^-^ availability at the nickel hydroxide surface during oxidation, promoting more extensive Ni(OH)_2_-to-NiOOH conversion under acoustic irradiation. This is supported be the negative shift of the cathodic reduction peak (ΔE_p,c_ ∼ −40 mV), in addition to reduced peak intensity, suggesting that the highly oxidized nickel species generated under acoustic irradiation are more stable and less readily reduced back to the resting divalent state during the reverse potential sweep.

Prior to establishing the thermally controlled protocol described in Section 2.1, a preliminary voltammetric screening was performed on a flat Ni electrode at higher frequencies (864 and 1146 kHz, direct configuration), which exhibited a qualitatively similar cathodic shift in the Ni(OH)_2_/NiOOH onset potential (**Fig. S11**). However, continuous long-term operation was exclusively standardized at 584 kHz (70% amplitude, indirect configuration) as it was the sole frequency allowing strict isothermal control of the bulk electrolyte while respecting the transducer's thermal limits. This finding is mechanistically distinct from previous applications of ultrasound to nickel-based electrocatalysis. Foroughi et al. investigated water splitting on Raney nickel under 408 kHz HFUS and on polycrystalline nickel under 24 kHz LFUS, reporting that ultrasound decreases operating overpotentials strictly through physical mechanisms – such as the rapid detachment of evolved gas bubbles to reduce bubble-gas overpotential or *ex situ* surface reconstruction. Crucially, in their *in situ* voltammetry and impedance analyses, the redox onset potential of the active nickel redox couple remained completely unaltered under sonication [21]. In contrast, the 450 mV cathodic shift in the Ni(OH)_2_/NiOOH transition observed in this work under stable high-frequency cavitation indicates that the acoustic field acts as a true electronic and kinetic modifier of the catalyst's solid-state chemistry. By dynamically perturbing the double layer as theorized by Pollet, the micro-acoustic field directly accelerates the coupled proton-electron deprotonation step required for the phase transition, enabling the active NiOOH oxidant state to be generated at significantly lower energy regimes [30]. To our knowledge, this is the first report of *in situ* ultrasound directly shifting the potential window over which a transition-metal oxyhydroxide redox couple becomes electrochemically active.

Improved bulk convection, macro-convective transport, and gas bubble detachment under ultrasonic fields are well documented [20], [31]. However, these purely physical hydrodynamic effects cannot fully account for the observed shift in the redox onset behavior and the resulting charge asymmetry. Instead, high-frequency acoustic cavitation induces intense, highly localized micro-convective perturbations directly at the electrode–electrolyte boundary layer. While low-frequency ultrasound (LFUS, 20 kHz) dominates bulk convective transport through violent, long-range transient cavitation, high-frequency stable cavitation operates on a fundamentally different spatial scale. Due to their micrometric resonant size (∼13 µm), the stable microbubbles generated at 584 kHz are capable of penetrating deep within the Nernst diffusion layer and the intricate 3D porous structure of the nickel foam ligaments without causing mechanical erosion [32], [33]. It is then proposed here that the continuous microstreaming and rapid interfacial fluid shear forces generated by these oscillating microbubbles significantly enhance the local accessibility and transport of co-reactant OH^-^ ions directly to the solid catalyst surface [20], [31]. This accelerated interfacial ionic supply dynamically perturbs the electrical double layer, facilitating the rate-determining adsorption of OH^-^ onto the nickel hydroxide framework [26], [30]. This localized micro-convection drastically lowers the activation energy barrier for the coupled proton-electron solid-state transition, shifting the active phase-generation onset potential cathodically. These results establish high-frequency acoustic cavitation as a non-invasive, dynamic lever to modulate interfacial charge-transfer kinetics, offering a paradigm complementary to conventional static catalyst design.

### 2.4 Glucose electrooxidation on activated Ni foam

Ultrasound-assisted glucose electrooxidation was systematically investigated in 0.10 M NaOH under temperature-controlled conditions (20.70 ± 1.90 °C) to eliminate bulk thermal contributions. CVs recorded in the absence and presence of acoustic irradiation (584 kHz, 70% amplitude) reveal a highly pronounced modification of the overall electrocatalytic response (Fig. 5**a**). In the blank supporting electrolyte (absence of glucose), the characteristic solid-state Ni(OH)_2_/NiOOH redox transition appears in the potential window of ∼1.40–1.50 V *vs.* RHE. Upon addition of 2.90 mM glucose under silent conditions, a distinct oxidation wave emerges at an onset potential of ∼1.30 V *vs.* RHE. This onset is consistent with a mediator-assisted catalytic pathway driven by the electrogenerated active Ni(III) oxyhydroxide phase [10], [34], [35].

**Fig. 5:**
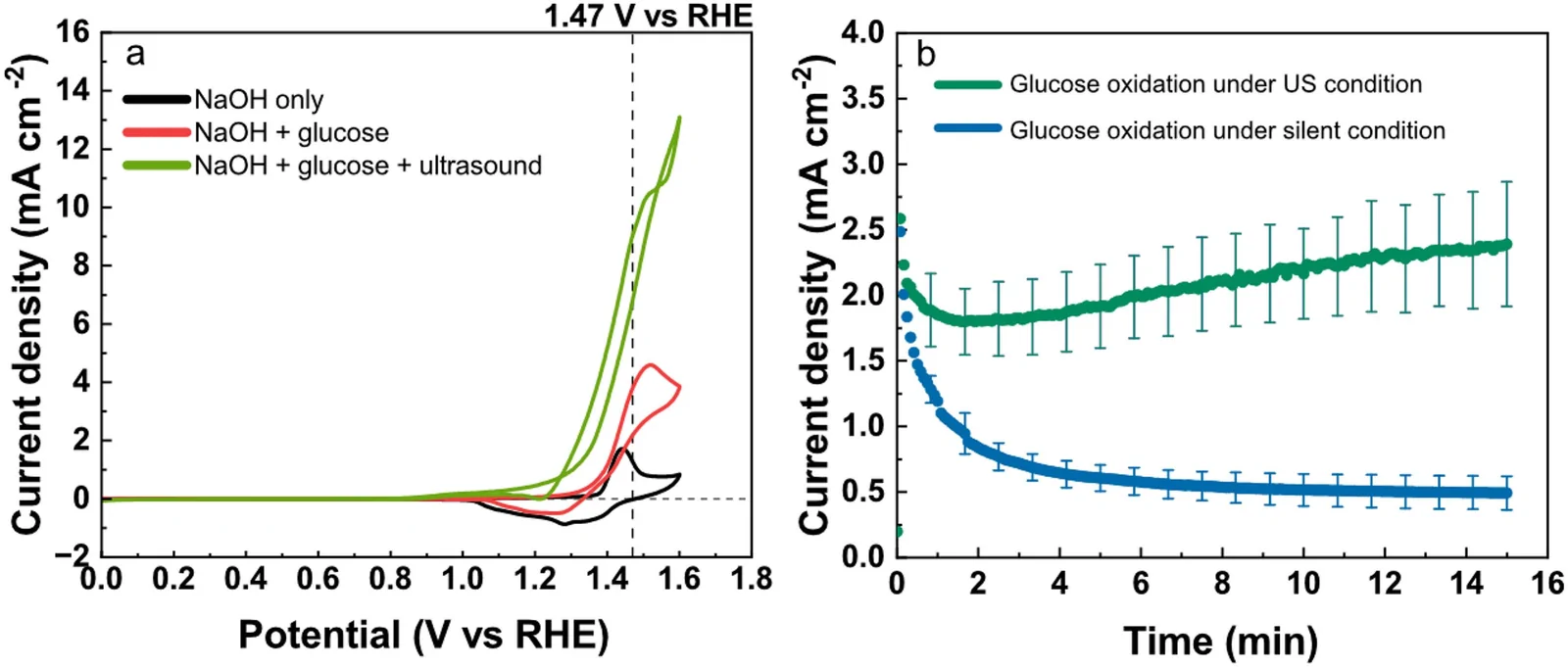
Ultrasound enhances NiOOH-catalysts for the glucose electrooxidation reaction on activated Ni foam. (a) CVs in 0.10 M NaOH recorded at 10 mV s^−1^ and 20.70 ± 1.90 °C: (black line) supporting electrolyte alone; (red line) in presence of 2.9 mM glucose (no ultrasound); (green line), in presence of 2.9 mM glucose with ultrasound (584 kHz, 70 % amplitude; indirect configuration). The dashed line marks the potential of 1.47 V *vs*. RHE used for chronoamperometry (CA); (b) CA at 1.47 V *vs*. RHE for 15 min (same electrolyte and temperature).

Strikingly, the application of HFUS causes a massive increase in the anodic current density, accompanied by a dramatic cathodic shift in the apparent onset of glucose electrooxidation down to ∼0.90–1.00 V *vs.* RHE (Fig. 5**a**). This early activation demonstrates that the catalytically active NiOOH surface sites become electrochemically accessible at significantly lower potentials under the influence of the high-frequency acoustic field. As a direct result, glucose electrooxidation proceeds efficiently under potential regimes where the silent electrode remains completely inactive. Although this applied potential range overlaps with the thermodynamic region of the competing OER, the voltammetry data show no evidence of oxygen gas evolution, confirming the high selectivity and preferential oxidation of glucose under these reaction conditions.

To assess whether these effects arise from permanent electrode modification, CVs were recorded before, during, and after short-duration ultrasound exposure, both in the indirect configuration (**Fig. S6**) and, separately, in the direct configuration without electrolyte temperature control (**Fig. S7**). In both cases, the redox current densities and peak potentials were almost completely restored upon cessation of ultrasound. This indicates that, over the timescale tested, the observed electrocatalytic enhancement is not associated with a persistent change in the electrode's electrochemical signature. This conclusion is based on electrochemical reversibility alone; it does not exclude slow, cumulative structural or compositional change that a short-timescale voltammetry comparison would not detect, nor was independent post-exposure structural characterization (e.g., SEM/EDS) performed specifically after ultrasound exposure. Extended-duration operation together with post-test surface characterization (XPS, Raman, etc.) would be required to fully exclude such effects.

The pronounced current enhancement achieved in the 1.00–1.40 V *vs.* RHE region cannot be rationalized solely by improved mass transport. Within this potential range, the solid-state Ni(II)/Ni(III) phase transition is fundamentally rate-limited by the interfacial diffusion/adsorption of hydroxyl ions OH^-^ and subsequent solid-state charge-transfer kinetics [11], [12], [36]. High-frequency acoustic cavitation successfully modulates these interfacial kinetics through three synergistic effects: (i) enhanced electrolyte penetration deep within the porous 3D foam structure to maximize electrochemically active surface area; (ii) continuous micro-convective disruption of the electrical double layer to accelerate OH^-^ diffusion/adsorption kinetics; and (iii) high localized shear forces that prevent surface passivation by sweeping away reaction intermediates. To further decouple these interfacial kinetic effects from simple hydrodynamic mass transport, comparative experiments were performed under LFUS (20 kHz; **Fig. S8**). Although LFUS also enhances current densities, the effect is restricted to higher potentials (∼1.40–1.60 V *vs.* RHE) and does not shift the onset potential. This behavior is typical of purely bulk solution diffusion-limited mass-transport enhancement. As summarized in Table 1, this contrast highlights a fundamental mechanistic divergence: while LFUS acts primarily as a physical mass-transport promoter, HFUS serves as an interfacial kinetic activator by modulating the redox transition itself [37], [38].

**Table 1:** Comparison of glucose electrooxidation onset potentials on activated Ni foam in 0.10 M NaOH.

Acoustic condition	Frequency	Amplitude	Configuration	Apparent onset potential (V *vs*. RHE)
Silent	N/A	N/A	Control	∼ 1.30
HFUS	584 kHz	70 %	Indirect (thermally controlled)	∼ 0.90 – 1.00
LFUS	20 kHz	20 %	Direct immersion	∼ 1.30

As introduced in Section 2.3, this heterogeneous, interfacial mode of activation contrasts with two related but mechanistically distinct precedents. Neha et al. [23] investigated glucose electrooxidation on a polycrystalline platinum electrode under low-frequency sonication (45 kHz); low-frequency ultrasound did not alter the onset potential of glucose oxidation in that study, with current enhancement restricted to the mass-transport-limited high-potential region – a behavior consistent with our own 20 kHz LFUS control experiments. Separately, Amaniampong et al. achieved selective oxidation of glucose to glucuronic acid under high-frequency ultrasound (550 kHz) in a catalyst-free, homogeneous aqueous system, via bulk radical sonolysis driven by hotspot bubble collapse rather than any heterogeneous electrode process [24]. No hydrogen evolution is observed under HFUS at open circuit in the present study (**Fig. S10**), confirming that direct sonolytic water splitting does not occur under these conditions; because anodic oxidation products were not independently identified or quantified in this work, we cannot fully exclude a minor contribution from bulk-phase radical chemistry to glucose conversion, but the potential-dependent onset behavior observed here, activity confined to the vicinity of the NiOOH redox couple rather than occurring uniformly across the sonicated volume, is consistent with a predominantly heterogeneous, mediator-assisted pathway on the acoustically activated Ni foam surface.

Chronoamperometric measurements performed at 1.47 V *vs.* RHE underscore the crucial role of HFUS in maintaining catalytic stability over time (Fig. 5**b**). Under silent conditions, the anodic current density undergoes a rapid and severe decay, stabilizing at ∼0.50 mA cm^-2^ within 15 min. This deactivation is well known to arise from the strong adsorption of carboxylic acid reaction intermediates and byproducts that block active surface sites on nickel in alkaline media [39]. In sharp contrast, under continuous high-frequency sonication, the current density remains remarkably stable and gradually climbs to ∼2.10 mA cm^-2^, yielding a sustained current enhancement factor of approximately 3.5 to 4 (calculated via Eq. (2), Fig. 6). This performance demonstrates that the localized microstreaming effectively suppresses surface passivation by continuously sweeping away adsorbed reaction intermediates or byproducts and evolved microbubbles from the pore structures. To quantitatively evaluate this enhancement, the chronoamperometric curves were integrated over the 900 s polarization period to determine the cumulative Faradaic charge. After subtracting the blank background contribution, the net Faradaic charge increased from 0.215 C under silent conditions to 0.855 C under HFUS. This corresponds to a nearly fourfold enhancement in the total charge transfer (ratio ∼ 3.98, calculated via Eq. (3)). This sustained charge capacity confirms that the high-frequency acoustic field does not merely provide a transient boost to the initial reaction rate, but actively prevents progressive catalyst passivation, thereby sustaining high-rate glucose electrooxidation over the tested operational timescale.

**Fig. 6:**
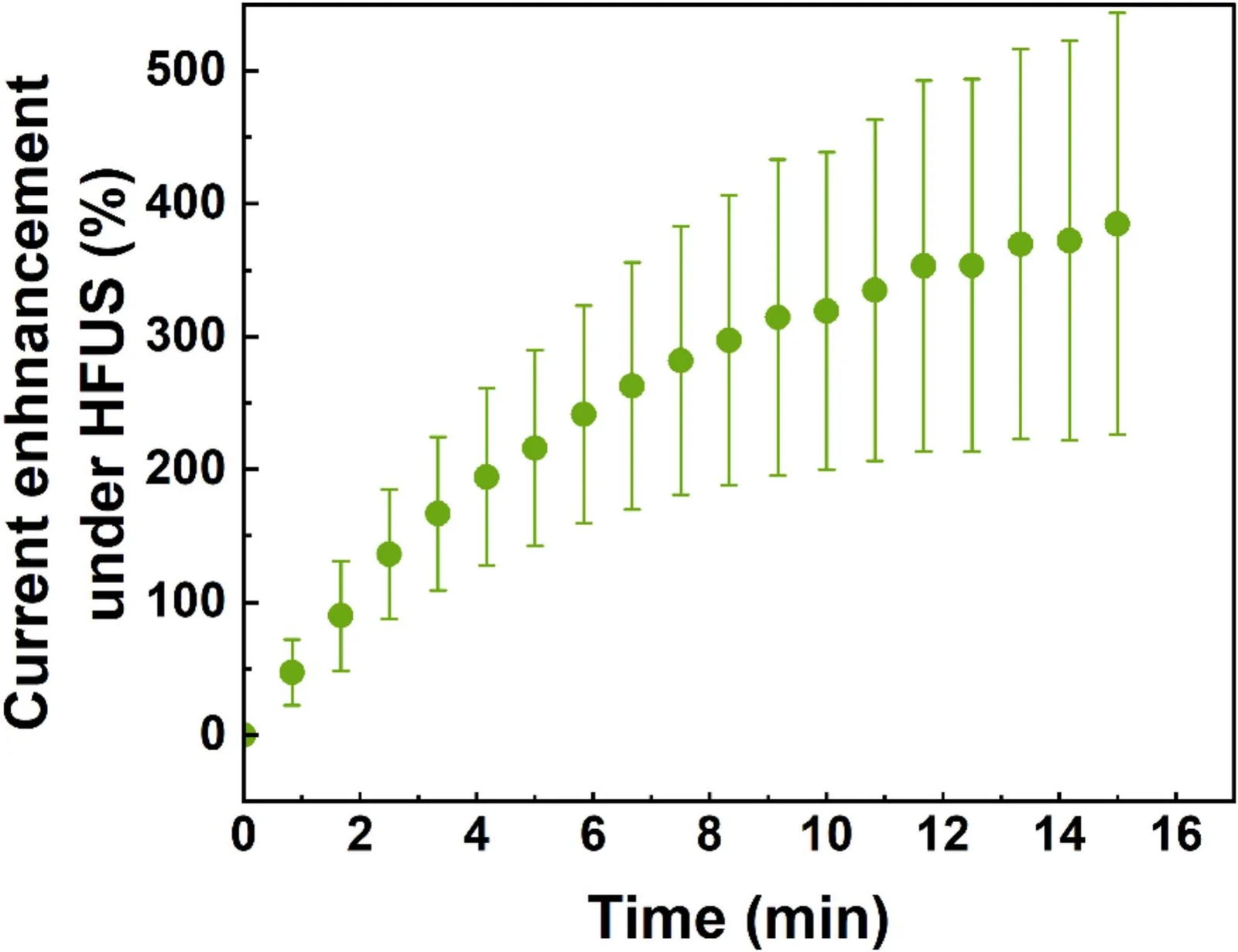
Evolution of the relative current enhancement under ultrasonic irradiation during 15 min of chronoamperometry at 1.47 V *vs.* RHE.

### 2.5 Coupled enhancement of hydrogen production via acoustic electroreforming

To evaluate the cathodic performance and demonstrate the practical viability of this high-frequency sonoelectrochemical reforming process, we monitored the HER at the counter electrode during chronoamperometry at a fixed anodic potential of 1.47 V *vs.* RHE. To avoid anodic substrate depletion and suppress any competing OER over the continuous 140 min electrolysis period, the initial glucose concentration was increased to 30 mM (representing a ten-fold increase compared to our analytical cyclic voltammetry experiments). Two essential control experiments were performed to verify the electrochemical origin of the gaseous products, as confirmed by the raw chromatograms (**Fig. S10**): first, no anodic current or hydrogen gas was detected in the absence of glucose (Fig. 7**a and 7b, black squares**); second, no hydrogen was generated under continuous high-frequency sonication in the absence of an applied potential (open circuit conditions, Fig. 7**b, green triangles**). These controls confirm that the high-frequency acoustic field alone does not drive direct sonolytic water splitting under these mild conditions, and that active anodic glucose oxidation is strictly required to sustain cathodic hydrogen generation.

**Fig. 7:**
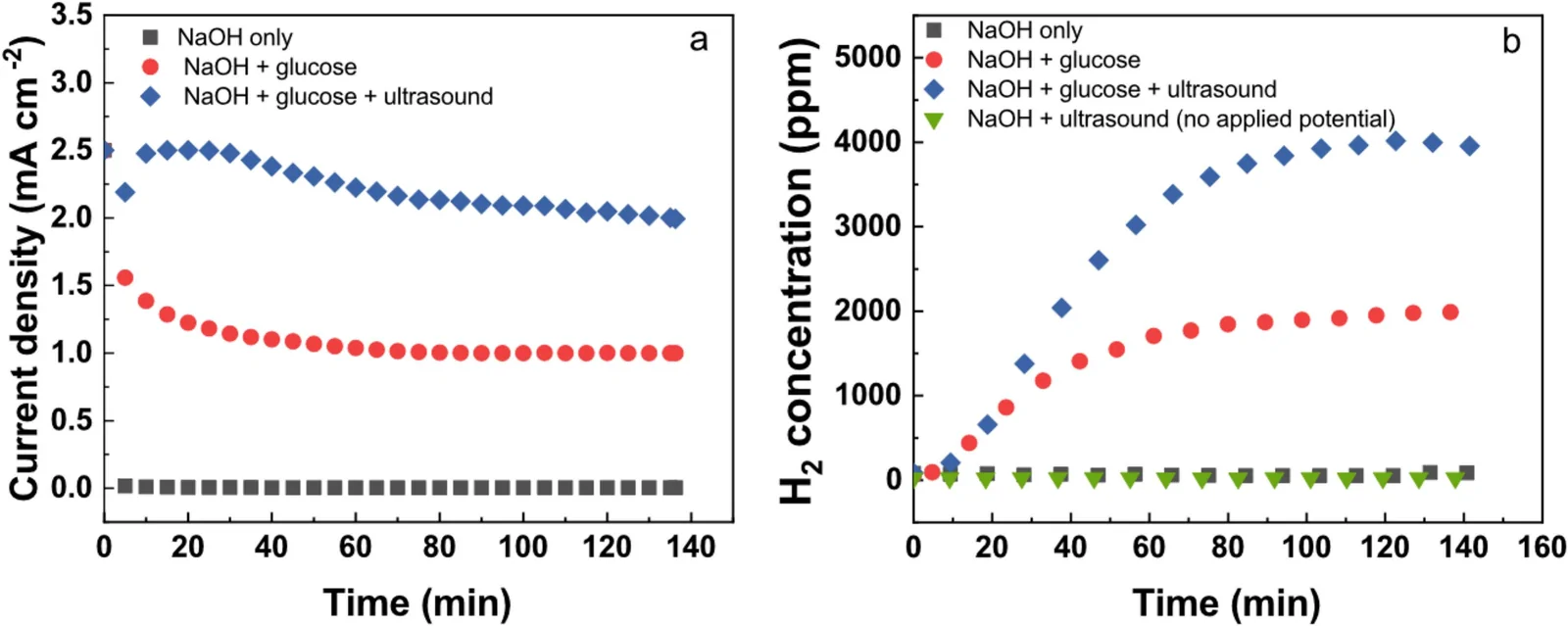
Coupled enhancement of anodic current and cathodic hydrogen evolution by ultrasound during electroreforming, (a) Chronoamperometry at 1.47 V *vs.* RHE in 0.10 M NaOH (black squares), NaOH + glucose (red circles), and NaOH + glucose with ultrasound irradiation (blue diamonds), (b) Time evolution of the integrated H_2_ peak area measured by micro-GC under the same conditions. A control experiment performed in NaOH under ultrasound irradiation without applied potential is also shown (green triangles) Ultrasound was applied at 584 kHz and 70 % amplitude. The integrated H_2_ peak area is normalized by a factor of 10^3^.

In the presence of glucose under silent conditions, the anodic current density remains stable at approximately 1.0 mA cm^-2^ (Fig. 7**a, red circles**), accompanied by a gradual and linear accumulation of hydrogen at the cathode (Fig. 7**b, red circles**). Upon the application of HFUS (584 kHz, 70% amplitude), both the anodic current density and the rate of cathodic hydrogen evolution experience a dramatic and immediate enhancement. The anodic current density doubles to approximately 2.0 mA cm^-2^ and remains remarkably stable throughout the continuous 140 min run (Fig. 7**a, blue diamonds**). In close agreement with this anodic enhancement, the cumulative volume of generated hydrogen under HFUS reached approximately twice the volume recorded under silent conditions, as monitored by real-time online micro-gas chromatography (micro-GC) (Fig. 7**b, blue diamonds**). By looking at how gas builds up over time, we can divide the process into two clear phases:

• During the initial linear stage, from t = 4.72 to 32.92 min under silent conditions and from t = 4.72 to 65.97 min under HFUS, hydrogen production proceeds at its maximum rate. The production rate increases from ∼38.4 ppm min^-1^ (silent) to ∼52.8 ppm min^-1^ (HFUS), reflecting sustained reactant availability and enhanced interfacial charge transfer.
• Beyond this active window, from ∼32.92 min to the end 140 min under silent conditions, and from ∼65.97 min to 140 min under HFUS, the hydrogen evolution curves transition into a steady plateau.

The active phase happens when glucose is plentiful at the electrode and the sound waves (microstreaming) stop the surface from getting blocked. The plateau happens later when glucose near the nickel foam starts to run out, and gas production matches the rate at which the micro-GC samples it. Because ultrasound keeps the active phase going twice as long as the silent test before reaching a flat line, it proves that acoustic cavitation helps overcome mass-transfer limits and keeps the catalyst working fast for a much longer time.

This direct correlation confirms that the acoustic activation of the anodic oxidation kinetics translates directly into an increased electron flux through the external circuit, which subsequently drives the enhanced reduction of protons at the cathode. Furthermore, the rapid microacoustic streaming and interfacial shear forces generated by the stable high-frequency cavitation bubbles significantly accelerate the detachment and removal of evolved hydrogen gas bubbles from the cathode surface [40]. In conventional water electrolysis, the accumulation of clinging gas bubbles blocks active catalyst sites and increases the local ohmic resistance (the “bubble effect” or “gas-blanketing” effect). Under HFUS, the continuous bubble sweep-out effect minimizes the bubble coverage and bubble overpotential, sustaining maximum electroactive surface area accessibility and enhancing the overall energy conversion efficiency

Quantitative analysis of the charge-to-product relationship strongly supports this coupled reforming mechanism. Integrating the chronoamperometric current transients over the entire 140 min period (after subtracting the blank background charge) reveals that the total Faradaic charge transferred under high-frequency ultrasound is approximately doubled compared to silent conditions. This twofold increase in cumulative electrical charge correlates precisely with the twofold increase in cumulative hydrogen volume detected by micro-GC. Collectively, these results demonstrate that high-frequency ultrasound successfully intensifies the overall electroreforming efficiency by dynamically coupling accelerated interfacial oxidation kinetics at the anode with enhanced hydrogen evolution at the cathode.

### 2.6 Limitations and strengths of the study

Although this study has limitations that define the boundaries of its conclusions:

(i) It focuses on a single model system (Ni foam/glucose); generalization to other catalysts or biomass-derived substrates has not been tested.
(ii) HFUS and LFUS were not operated under matched acoustic power, intensity, or cavitation yield, and were implemented in physically distinct reactor geometries (indirect plate transducer vs. direct immersion horn); a direct power-normalized comparison was not physically viable with the available hardware. The LFUS condition should therefore be read as a qualitative benchmark rather than a matched control.
(iii) Cavitation activity was quantified indirectly via H_2_O_2_ dosimetry, without real-time spatial mapping of acoustic pressure or bubble dynamics within the porous electrode.
(iv) NiOOH formation was quantified from faradaic charge integration rather than from direct structural or spectroscopic evidence. While charge-based quantification is an established approach for estimating relative changes in electroactive Ni oxyhydroxide content [11], [12], [36], it does not provide direct confirmation of the NiOOH phase itself. However, the electrochemistry of Ni-based materials is well known since several decades [41], and the Ni(OH)_2_/NiOOH phase transition with potential well documented [29]. Operando spectroscopic techniques such as Raman or X-ray absorption spectroscopy (XAS) would offer direct structural evidence, but are technically difficult to implement under active high-frequency cavitation: intense microstreaming and bubble activity at the electrode–electrolyte interface generate significant optical scattering for Raman, and the thin-window flow-cell geometries required for liquid-phase XAS/XPS are difficult to reconcile with the bulky piezoelectric transducer hardware used here.
(v) Reversibility was assessed only by electrochemical means over a short exposure timescale. The evidence for the dynamic, non-permanent nature of the HFUS effect (Figs. S6 and S7) consists of voltammetric comparisons before and after brief ultrasound exposure; no extended-duration durability testing was performed, and no independent structural or compositional characterization (SEM/EDS) was carried out specifically after ultrasound exposure, as distinct from the pre-/post-activation characterization shown in Figs. 2 and S3–S5. Electrochemical reversibility of the redox signature is consistent with, but does not on its own establish, the absence of slow, cumulative microstructural change. Extended operation combined with post-test SEM/EDS characterization is identified as a necessary next step.
(vi) Absolute faradaic efficiency toward hydrogen was not calculated. Quantifying FE(H_2_) requires converting micro-GC signal to an absolute molar quantity, which depends on the reactor headspace volume and gas-sampling regime (closed accumulation vs. continuously purged), parameters not fully characterized in the present study. We therefore report hydrogen evolution as a relative, charge-correlated quantity rather than an absolute efficiency: the ∼2-fold increase in cumulative Faradaic charge under HFUS closely matches the ∼2-fold increase in cumulative micro-GC H_2_ signal, indicating that the enhanced anodic charge is converted to cathodic hydrogen with comparable, rather than degraded, efficiency relative to silent conditions. Absolute FE(H_2_) quantification is identified as a priority for future work.
(vii) The electrical energy required to drive HFUS substantially exceeds the electrochemical power gained. Using the electrical input power reported for the transducer (∼155 W, continuous mode) and the anodic current/potential data from Section 2.5, the electrochemical power draw increases from ∼5.9 mW (silent) to ∼11.8 mW (HFUS), a net gain roughly four to five orders of magnitude smaller than the ultrasound input power. This estimate uses the anodic potential vs. RHE rather than the full two-electrode cell voltage, so the true electrochemical power is likely somewhat higher; this does not change the conclusion at this scale. The present laboratory-scale configuration is therefore not energy-competitive in absolute terms. The purpose of this study is to establish that acoustic cavitation can modulate interfacial redox chemistry as a mechanistic principle, not to demonstrate an energy-optimized process. Pulsed (duty-cycled) irradiation, reduced acoustic amplitude operated near the cavitation threshold, optimized transducer geometry, and reactor designs that localize acoustic energy at the electrode rather than the bulk electrolyte are expected to substantially reduce the parasitic power draw, and are identified as priorities for future process-engineering work.

This work nonetheless has strengths and brings new insights on the effect of combining electrochemistry and ultrasound for biomass activation and hydrogen production:

(i) A thermally decoupled indirect reactor configuration that isolates acoustic cavitation from bulk thermal artifacts, validated by both temperature monitoring and H_2_O_2_ dosimetry (Fig. 1);
(ii) A stable, cavitation regime at 584 kHz, consistent with the Minnaert equation's prediction of ∼13 µm resonant bubble diameters at this frequency, in line with direct observations of stable cavitation fields at comparable frequencies [18], [32] consistent with preservation of the porous nickel foam structure, as indicated by the reversibility of the redox response upon cessation of irradiation, over the exposure timescales tested (Figs. S6–S7);
(iii) Direct evidence of interfacial kinetic modulation, with a ∼450 mV shift in the NiOOH formation onset, rather than only current amplification;
(iv) A frequency-dependent qualitative comparison with LFUS that strengthens, rather than weakens, the interfacial origin of the HFUS effect: despite using a physically distinct LFUS configuration (direct immersion, close electrode–horn proximity, smaller cell volume) that would, if anything, be expected to favor a stronger physical/hydrodynamic artifact, no shift in the glucose oxidation onset potential was observed under LFUS. This contrast supports a frequency-specific, interfacial origin for the HFUS effect rather than a generic consequence of acoustic exposure.

## 3 Conclusion

This study demonstrates that high-frequency ultrasound (HFUS, 584 kHz) acts as a dynamic physical field that modulates the interfacial redox chemistry of nickel electrodes, enabling enhanced glucose electrooxidation under mild conditions. The main findings are summarized as follows:

• Kinetic modulation: acoustic irradiation induces a substantial cathodic shift of ∼450 mV in the Ni(OH)_2_/NiOOH redox onset potential. This lowers the activation barrier for generating the active NiOOH oxidant, allowing glucose electrooxidation to commence at significantly lower potentials.
• Anodic current enhancement: by dynamically perturbing the double layer to facilitate OH^-^ adsorption and continuously sweeping away reaction intermediates, HFUS prevents surface passivation and increases the steady-state anodic current density by approximately fourfold (∼400%).
• Coupled hydrogen production: the accelerated interfacial oxidation kinetics at the anode translate directly into improved system-level performance, resulting in a twofold (∼200%) enhancement in cumulative cathodic hydrogen gas evolution.
• Reversible dynamic activation: Over the exposure timescales tested, the acoustic enhancement is consistent with localized micro-convection and double-layer perturbation, with no evidence of permanent morphological destruction detected by electrochemical means. The electrochemical signature was fully restored upon cessation of irradiation, although this reversibility does not on its own exclude slow, cumulative microstructural changes beyond the tested timescale.

More broadly, this work highlights practical applications and future directions for high-frequency ultrasound in electrocatalysis. In terms of practical applications, this method demonstrates a mechanistic route for the electroreforming of biomass-derived molecules (such as glucose) to simultaneously produce hydrogen and valuable chemicals without using expensive noble metals. Looking ahead, future work should focus on using pulsed acoustic fields to reduce energy consumption.

## 4 Experimental

### 4.1 Materials and reagents

Commercial nickel foam (Goodfellow®, 99.5% purity, thickness 0.9 mm, porosity 93%, pore density of 20 pores per linear cm, corresponding to approximately 50 pores per inch (ppi)) was used as both the working and counter electrode substrate. For the electrolyte preparation, sodium hydroxide (NaOH, ≥ 99%, Sigma-Aldrich) and D-glucose (≥ 99%, Sigma-Aldrich) were used as received, without further purification. All aqueous solutions were prepared using ultrapure water (Milli-Q, Millipore, resistivity ≥ 18.2 MΩ·cm, hereafter referred to as UP water).

### 4.2 Electrode preparation and electrochemical activation

Nickel foam electrodes (geometric surface area = 0.50 cm^2^) served as the working electrode. A larger nickel foam (geometric surface area = 4.00 cm^2^) served as the counter electrode, and a reversible hydrogen electrode (RHE, HydroFlex, Gaskatel) as the reference electrode. For hydrogen quantification experiments, the working electrode area was increased to 4.00 cm^2^ to improve glucose conversion, and the counter electrode area was increased to 8.00 cm^2^. Before use, the Ni foam electrodes were rinsed with ultrapure water, then successively sonicated for 30 min in UP water, 15 min in 1.00 M HCl (in UP water), and again for 30 min in UP water. The electrodes were then dried under a flow of air.

All electrochemical measurements, including the initial electrochemical activation, cyclic voltammetry, and chronoamperometry, were performed using a Metrohm Autolab µAutolab Type III potentiostat, controlled via NOVA software.

The activation protocol consisted of four consecutive steps: (i) 20 cycles between 0.00 and 1.30 V *vs.* RHE at 100 mV s^-1^, (ii) 25 cycles between 0.80 and 1.60 V *vs.* RHE at 50 mV s^-1^, (iii) 10 cycles between 0.80 and 1.60 V *vs.* RHE at 20 mV s^-1^, and (iv) 10 cycles between 0.80 and 1.60 V *vs.* RHE at 10 mV s^-1^. Step (i) promotes surface hydroxylation of metallic Ni to Ni(OH)_2_. Steps (ii)–(iv) progressively extend the potential window for formation of the Ni(OH)_2_/NiOOH redox couple, to obtain a well-defined surface layer. All current densities reported in this work are normalized to the geometric surface area of the electrode. All nickel foam electrodes used in this study were subjected to the electrochemical activation protocol described above, except where explicitly stated otherwise (e.g., as-received Ni foam used for comparison, Fig. 3).

### 4.3 Sonoelectrochemical setup and temperature control

High-frequency ultrasonic irradiation was applied using a piezoelectric transducer (Meinhardt E/805/T/M, stainless-steel/titanium head, active diameter 75 mm). The experiments were conducted at a fixed frequency of 584 kHz. The generator amplitude was set to 70% of the nominal controller output (continuous mode), corresponding to a continuous electrical input power of 155 W, as monitored on the digital controller.

Two experimental configurations were evaluated (Fig. 8): (i) a direct configuration, in which the electrolyte was in direct contact with the transducer, and (ii) an indirect configuration. To ensure identical baseline conditions, the electrolyte volume (50 mL) and cell geometry were maintained constant. To isolate the physical and chemical effects of acoustic cavitation from bulk thermal convective artifacts, all primary sonoelectrochemical measurements were systematically performed using the indirect ultrasonication configuration.

**Fig. 8:**
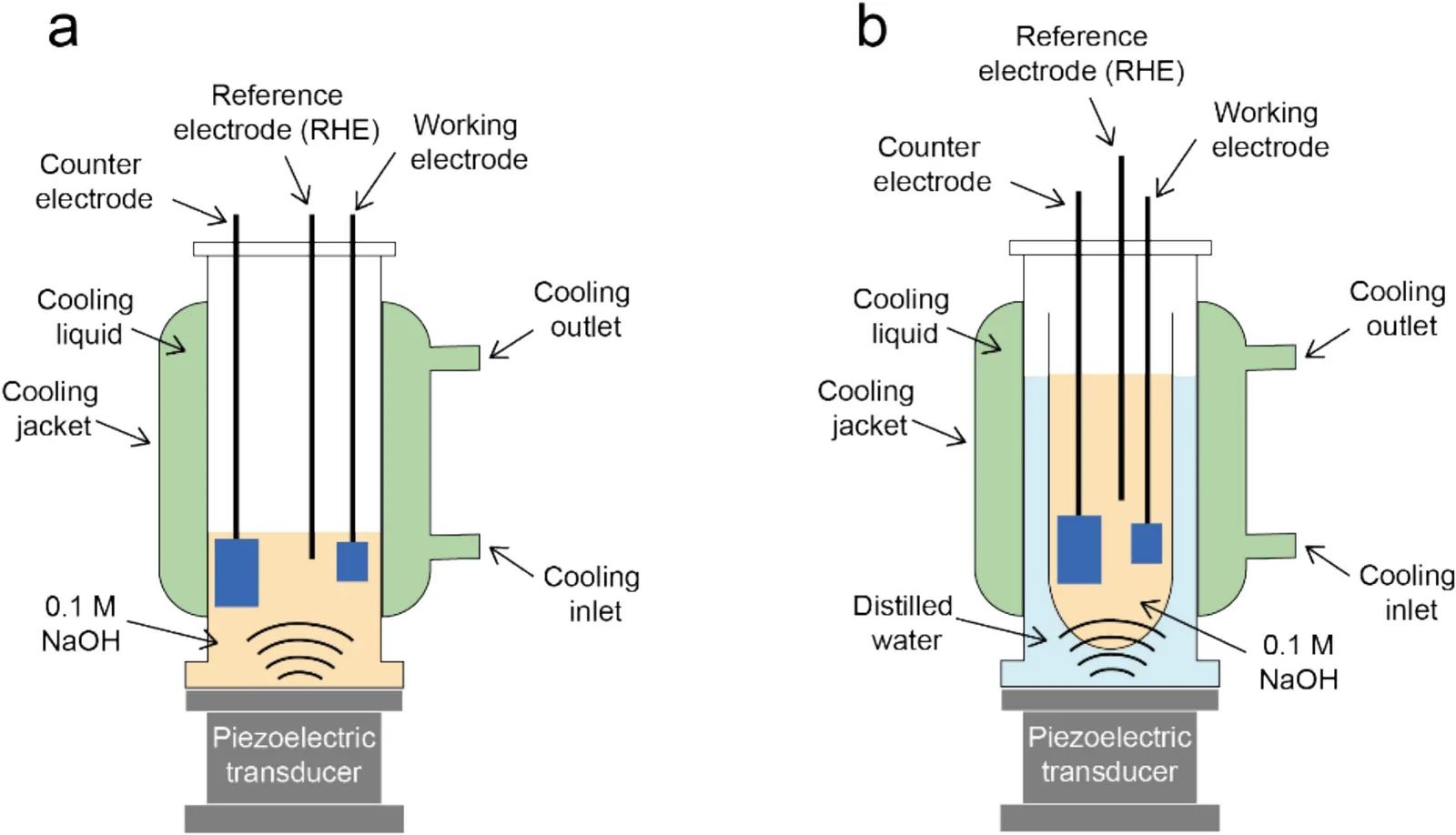
(a) a double-walled high-frequency transducer-based sonoelectrochemical cell operated in direct ultrasonication mode, and (b) a double-walled high-frequency transducer-based sonoelectrochemical cell operated in indirect ultrasonication mode. The temperature was monitored using a thermocouple immersed in the electrolyte.

As illustrated in the experimental setup **(**Fig. 8), this configuration is based on a nested reactor design. The working, reference, and counter electrodes are immersed in a smaller inner electrochemical cell containing 50 mL of the 0.10 M NaOH electrolyte. This inner cell is securely positioned inside a larger outer glass reactor filled with 150 mL of distilled water. The piezoelectric transducer is mounted at the base of the outer reactor, and the distance between the electrode and transducer is approximately 2 cm. In this indirect mode, the distilled water acts as an acoustic coupling medium, transmitting the high-frequency sound waves from the transducer face through the glass wall of the inner cell to generate cavitation within the electrolyte.

To prevent the intense heat generated at 584 kHz, the setup uses two cooling methods. First, the outer glass reactor is equipped with a cooling jacket circulating a liquid coolant. Second, the transducer base sits on an active cooling plate (-2 to 0 °C circulating coolant). This dual-cooling strategy continuously extracts heat from the coupling medium.

To conduct all experiments at a comparable temperature, the reactor and electrolyte were first cooled to approximately 7 °C using an external chiller circulating coolant at −2 to 0 °C. Ultrasound was then applied in the absence of electrodes, allowing the electrolyte temperature to rise and stabilize at 20.70 ± 1.90 °C under continuous irradiation. Electrodes and glucose were introduced only after this thermal stabilization, with ultrasonic irradiation maintained throughout. This protocol successfully stabilized the bulk electrolyte temperature, ensuring that the electrochemical measurements, which are highly sensitive to temperature, were conducted under identical thermal conditions during both silent and ultrasonic experiments.

### 4.4 Low-frequency ultrasound

Low-frequency ultrasound was applied using a 20 kHz ultrasonic horn (direct sonication mode, axial immersion). As detailed in **Fig. S8**, the reactor was operated in a direct sonication mode. The ultrasonic horn was immersed axially into the electrochemical cell containing 50 mL of the 0.10 M NaOH electrolyte, alongside the nickel foam working electrode, counter electrode, and a Luggin capillary connecting the reference electrode to the bulk electrolyte. The ultrasound amplitude was set to 20%. To mitigate the intense heat dissipation typical of direct low-frequency cavitational collapse, the cell is equipped with a double-walled cooling jacket circulating coolant maintained at 0 °C.

Due to the fundamental differences in reactor geometry (indirect plate transducer vs. direct immersion horn) and cavitation bubble dynamics, a direct matching of acoustic power density between the high- and low-frequency setups was not physically viable. Instead, the 20 kHz setup was employed strictly as a qualitative benchmark. Its primary purpose was to investigate the aggressive mechanical impact of low-frequency transient cavitation on the surface integrity and mass transport profile of the nickel foam working electrode, contrasting it with the gentler stable cavitation regime achieved at 584 kHz.

### 4.5 Chemical dosimetry of cavitation

Acoustic cavitation activity and the rate of radical generation were quantified via chemical dosimetry using titanium(IV) oxysulfate (TiOSO_4_) colorimetric method, adapted from the standard sonochemical calibration principles described by Iida et al. and Manickam et al. The colorimetric reagent was prepared by dissolving 0.52 g of solid TiOSO_4_ in deionized water under magnetic stirring. Subsequently, 2.80 mL of concentrated sulfuric acid (H_2_SO_4_, 18 M) was slowly added to the solution, and the mixture was diluted with deionized water in a volumetric flask to a final volume of 100 mL. Quantitative calibration was performed using a commercial 30% w/v H_2_O_2_ stock solution, which was diluted with deionized water to obtain a standard concentration range of 0.0005 to 0.005 M.

During sonochemical experiments, 0.5 mL aliquots of the bulk electrolyte were sampled every 30 minutes over a 120 minutes irradiation period. Each sample and calibration standard was directly mixed with 0.5 mL of the TiOSO_4_ reagent in a quartz cuvette. Absorbance of the resulting yellow pertitanic acid complex was measured at 412 nm using a UV-Vis spectrophotometer. The calibration produced a linear fit (R^2^ = 0.9991) with a molar extinction coefficient of 388 M^-1^ cm^-1^. The absolute H_2_O_2_ concentration in the reactor was calculated using the dilution-corrected linear regression defined in Eq. (1):

(1) $$C=2\times (\frac{A-0.006}{388.00})$$

Where A is the absorbance at 412 nm.

The chemical dosimetry measurement was performed as a single representative run to evaluate the baseline radical generation.

### 4.6 Electrochemical measurements

Glucose electrooxidation was investigated in 0.10 M NaOH. For voltammetric studies, a 2.9 mM glucose concentration was obtained by adding 1.50 mL of a 0.10 M glucose solution to 50 mL of the supporting electrolyte (yielding a final volume of 51.50 mL). Cyclic voltammograms were recorded within a potential window of 0.00–1.60 V *vs.* RHE at a scan rate of 10 mV s^-1^, under both silent and ultrasound conditions. Voltammetric onset potentials were determined using the tangent method, defined graphically as the intersection of the extrapolated baseline with the tangent line drawn at the point of maximum slope of the anodic wave.

Chronoamperometry measurements were performed at a fixed potential of 1.47 V vs. RHE, under silent and ultrasound conditions. Current densities and charges were normalized to the geometric surface area of the working electrode. Where applied, currents and charges were corrected by subtracting the corresponding blank (supporting-electrolyte-only) response, recorded under otherwise identical conditions.

For hydrogen quantification experiments, the glucose concentration was increased to 30 mM, to avoid substrate depletion during prolonged chronoamperometric operation. Percentage enhancement under HFUS relative to silent conditions was calculated as Eq. (2):

(2) $$Enhancement\left(\%\right)=\frac{j_{\mathrm{HFUS}}-j_{\mathrm{silent}}}{j_{\mathrm{silent}}}\times 100$$

where j is the geometric current density (mA cm^-2^). Enhancement was evaluated at each time point.

The cumulative charge enhancement was similarly expressed as Eq. (3):

(3) $$\mathrm{Ratio}=\frac{Q_{\mathrm{HFUS}}}{Q_{\mathrm{silent}}}$$

where Q is the Faradaic charge (C) obtained by integration of the chronoamperometric current over the stated time window. Chronoamperometric measurements and the resulting enhancement values were determined in triplicate (n = 3).

### 4.7 Gas analysis

To couple anodic glucose electroreforming with cathodic hydrogen production, the initial glucose concentration was increased to 30 mM to avoid mass-transport limitations during 140 minutes of chronoamperometric experiments. Hydrogen evolution was quantified using an Inficon Micro GC Fusion micro gas chromatograph (micro-GC), on-line during electrolysis. The instrument comprises two columns: Column A (Rt-Msieve 5A, 30 m length, 0.53 mm internal diameter, 50 µm film thickness) for the separation and detection of H_2_, O_2_, N_2_, CH4, and CO; Column B (Rt-Q-BOND, 30 m length, 0.53 mm internal diameter, 20 µm film thickness) for air, CH_4_, CO_2_, C2H_6_, C_2_H_4_/C_2_H_2_, C_3_H_8_, C3H_6_, C_4_–C_8_ hydrocarbons, and H_2_S detection. Calibration was performed using a certified gas mixture (**Fig. S9**).

Gas samples were withdrawn directly from the sealed headspace of the electrochemical cell; no continuous purge or carrier gas flow was applied to the reactor. Headspace gas injections into the micro-GC were performed automatically and continuously throughout each chronoamperometric run, with successive analyses spaced approximately 4 min 40 s to 4 min 45 s apart (mean ∼ 4.7 min), corresponding to the fixed analytical cycle time of the two-column micro-GC method. To prevent carryover contamination and maintain maximum system cleanliness, gas quantification runs were conducted as single experiments performed on dedicated experimental days.

### 4.8 SEM and EDS characterization

Surface morphology of nickel foam before and after electrochemical activation and catalytic testing was characterized by scanning electron microscopy (SEM) using an FEI Teneo VolumeScope microscope. The instrument operated at an accelerating voltage of 20 kV and a working distance of 10 mm. Elemental mapping and chemical surface compositions were obtained using an Oxford X-Max 150 energy-dispersive X-ray spectrometer (EDS).

### CRediT authorship contribution statement

**Youness Boukarkour:** Writing – review & editing, Writing – original draft, Methodology, Investigation, Formal analysis. **Christophe Coutanceau:** Writing – review & editing, Supervision, Resources, Funding acquisition, Formal analysis, Conceptualization. **Karine.De Oliveira Vigier:** Writing – review & editing, Supervision, Project administration, Funding acquisition, Conceptualization. **Teko Wilhelmin Napporn:** Writing – review & editing, Supervision, Resources, Project administration, Formal analysis, Conceptualization. **Prince Nana Amaniampong:** Writing – review & editing, Validation, Supervision, Project administration, Funding acquisition, Conceptualization.

## Declaration of competing interest

The authors declare that they have no known competing financial interests or personal relationships that could have appeared to influence the work reported in this paper.

## Appendix A Supplementary data

Supplementary Data 1 Supporting Information detailing experimental setup, characterizations, cyclic voltammograms and gaseous phase analysis using micro-GC.
