# Supplementary material for: High-frequency ultrasound modulates the interfacial Ni(OH)2/NiOOH redox transition to enhance glucose electrooxidation and hydrogen production
Source: Ultrason Sonochem. 2026 Sep 28;134:108085. doi: 10.1016/j.ultsonch.2026.108085 (PMC13654560; doi:10.1016/j.ultsonch.2026.108085)
Supplement: Supplementary file 1 [file mmc1.docx]

**High-Frequency Ultrasound Modulates the Interfacial Ni(OH)_2_/NiOOH Redox Transition to Enhance Glucose Electrooxidation and Hydrogen Production**

Youness Boukarkour^1^, Christophe Coutanceau^1,2^, Karine. De Oliveira Vigier, Teko Wilhelmin Napporn^1,2, *^, Prince Nana Amaniampong^1, *^

^1^Institut de Chimie des Milieux et Matériaux de Poitiers, University of Poitiers, CNRS, 1 Rue Marcel Doré, 86073 Poitiers, France

^2^French Research Network on Hydrogen (FRH2) of CNRS, Nantes, France

*Corresponding authors: [prince.nana.amaniampong@univ-poitiers.fr](mailto:prince.nana.amaniampong@univ-poitiers.fr); [teko.napporn@univ-poitiers.fr](mailto:teko.napporn@univ-poitiers.fr)

**Figure S1:** Schematic representation and photographic views of the indirect high-frequency sonoelectrochemical reactor used for glucose electrooxidation experiments. The setup includes a three-electrode electrochemical cell (Ni foam working electrode, reference electrode vs RHE, counter electrode), mounted above a 584 kHz piezoelectric transducer with controlled cooling (0 °C circulating coolant) to minimize thermal effects.

**Figure S2:** Cyclic Voltammograms of Ni foam in 0.1 M NaOH (no ultrasound) to activate Ni foam at (22 ± 2 °C); (a) step 1: 0-1.30 V vs RHE, 20 cycles recorded at 100 mV s^-1^; (b) Step 2: 0.80-1.60 V vs RHE, 25 cycles recorded at 50 mV s^-1^; (c) Step 3: 0.80–1.60 V, 10 cycles recorded at 20 mV s^-1^; (d) Step 4: 0.80–1.60 V, 10 cycles recorded at 10 mV s^-1^.

**Figure S3:** Scanning electron microscopy (SEM) image of Ni foam before and after electrochemical activation. (a) a global view SEM image of the as-received Ni foam showing smooth ligaments and an open porous structure. (b) a global view SEM image after the electrochemical activation of as the as-received Ni foam. (c) High-magnification view of the as -received foam (region highlighted in a), revealing a relatively smooth surface. (d) ) High-magnification view of the activated foam (region highlighted in a), showing increased surface roughness.

**Figure S4:** SEM and EDX characterization of as-received Ni foam. (a) High-magnification SEM image of pristine Ni foam surface. (b) EDX elemental map of Ni (Kα).(c) The corresponding EDX spectrum showing dominant Ni peaks and absence of significant oxygen signal.

**Figure S5**: SEM and EDX characterization of electrochemically activated Ni foam. (a) High-magnification SEM image after activation, showing surface roughening. (b) EDX elemental map of oxygen (O Kα) indicating uniform oxygen distribution. (c) EDX elemental map of Ni (Kα). (d) Corresponding EDX spectrum showing the appearance of an oxygen peak and co-localization with Ni, consistent with formation of a Ni(OH)₂/NiOOH surface layer.

**Figure S6:** Cyclic voltammograms of activated Ni foam in 0.10 M NaOH in presence of recorded of 2.91 mM glucose recorded at 10 mV s^-1^: (a) Before HFUS (black); (b) during HFUS irradiation 584 kHz, 70% amplitude (red); and after ultrasound was stopped (blue).

**Figure S7:** Cyclic voltammograms recorded on a 2 cm^2^ Ni foam electrode in 0.10 M NaOH in the direct sonoelectrochemical configuration, and without controlling the electrolyte temperature: (a) CVs recorded at 10 mV s^-1^ before ultrasound (Blank, dotted line) and under HFUS irradiation at 584 kHz with amplitudes of 70% (blue) and 100% (orange); (b) comparison of the voltammograms before ultrasound (dotted line) and after ultrasound irradiation was stopped (red).

**Figure S8**: Sonoelectrochemical reactor and electrochemical response of glucose oxidation under low-frequency ultrasound (20 kHz) in 0.10 M NaOH aqueous solution. (a) Schematic representation and photographs of the low-frequency reactor equipped with an ultrasonic probe, a nickel foam electrode (geometric area 0.5 cm^2^), a nickel foam counter electrode (geometric area 4 cm^2^), and a reference electrode (RHE) connected through a luggin capillary. The reactor contains 50 mL of 0.10 M NaOH electrolyte and surrounded by a double wall cooling jacket with circulating coolant maintained at 0°C. (b) cyclic voltammograms recorded at 10 mV s^-1^ in the absence of glucose (black), in the presence of 2.9 mM glucose (blue), and with glucose under ultrasound irradiation (red, 20 kHz, 20% amplitude). (c) chronoamperometric response at 1.47 V vs. RHE in 0.10 M NaOH containing 2.9 mM glucose for 30 min, recorded in silent conditions (blue) and under ultrasound irradiation (red, 20 kHz, 20% amplitude)

**Figure S9:** Micro-GC calibration chromatogram obtained using a certified gas mixture containing 100 ppm H_2_, 500 ppm CH_4_, and 500 ppm CO balanced in air (20.9% O_2_, 78.79% N_2_). (a) Full chromatogram showing three retention time regions; (b) Region 1: H_2_ detection; (c) Region 2: O_2_ and N_2_ separation; (d) Region 3: CH_4_ and CO detection.

**Figure S10 :** Overlay of representative micro-GC chromatograms corresponding to hydrogen detection under different experimental conditions: electrochemistry without glucose (black), electrochemical glucose oxidation under silent conditions (red), electrochemical glucose oxidation under ultrasound irradiation (blue), and ultrasound treatment without electrochemical polarization (green). The enhanced H₂ peak intensity under coupled electrochemistry and ultrasound confirms the increase in hydrogen production observed in the main text.

**Figure S11: Cyclic voltammograms of a flat nickel electrode (2 cm^2^, counter electrode Ni foam 8 cm^2^) in 0.1 M NaOH under ultrasonic irradiation at (a) 584 kHz, (b) 864 kHz and (c) 1146 kHz, recorded at 10 mV s^-1^ between 0 and 1.6 V vs. RHE at amplitudes of 0 (silent), 20, 50, 70 and 100%. Chiller setpoint 0 °C. Note that stable thermal control was achieved only at 584 kHz; at 864 and 1146 kHz the electrolyte temperature was not stabilised within the transducer's 40 °C operating limit, and these datasets therefore contain an uncontrolled thermal contribution and are presented for qualitative screening purposes only.**
